# The importance of dynamic ultrasound in the diagnosis of tibialis anterior muscle herniation

**DOI:** 10.1186/s13089-014-0014-0

**Published:** 2014-09-04

**Authors:** Suheil Artul, George Habib

**Affiliations:** 1 Radiology Department, EMMS Nazareth Hospital, Nazareth 16100, Israel; 2 Faculty of medicine, Bar Ilan University, Nazareth 16100, Israel; 3 Rheumatology clinic, EMMS Nazareth Hospital, Nazareth 16100, Israel

**Keywords:** Muscle hernia, Ultrasound, Tibialis anterior

## Abstract

Tibialis anterior muscle hernia is a challenging diagnosis. Ultrasound findings generally are negative because patients come to ultrasound study from home when they are at rest. When the operator of ultrasound suspects a muscle hernia, he has to scan the affected limb or the affected organ dynamically at rest and after stressing the limb. Here, we present a case of a 19-year-old dancer with anterior leg mass with negative ultrasonographic findings at rest but was diagnosed with tibialis anterior hernia after stress dynamic ultrasound.

## Background

The tibialis anterior muscle is the most common site of muscle herniation. It presents clinically as soft tissue mass, usually indolent, but some patients have pain during limb movements. The mass can disappear during rest. This behavior of the mass is the clue for suspecting this entity. Imaging plays a crucial role in diagnosis. MRI can be of help but because of its on-off appearing, this makes dynamic ultrasound the gold standard of examination.

## Case presentation

**Figure 1:**
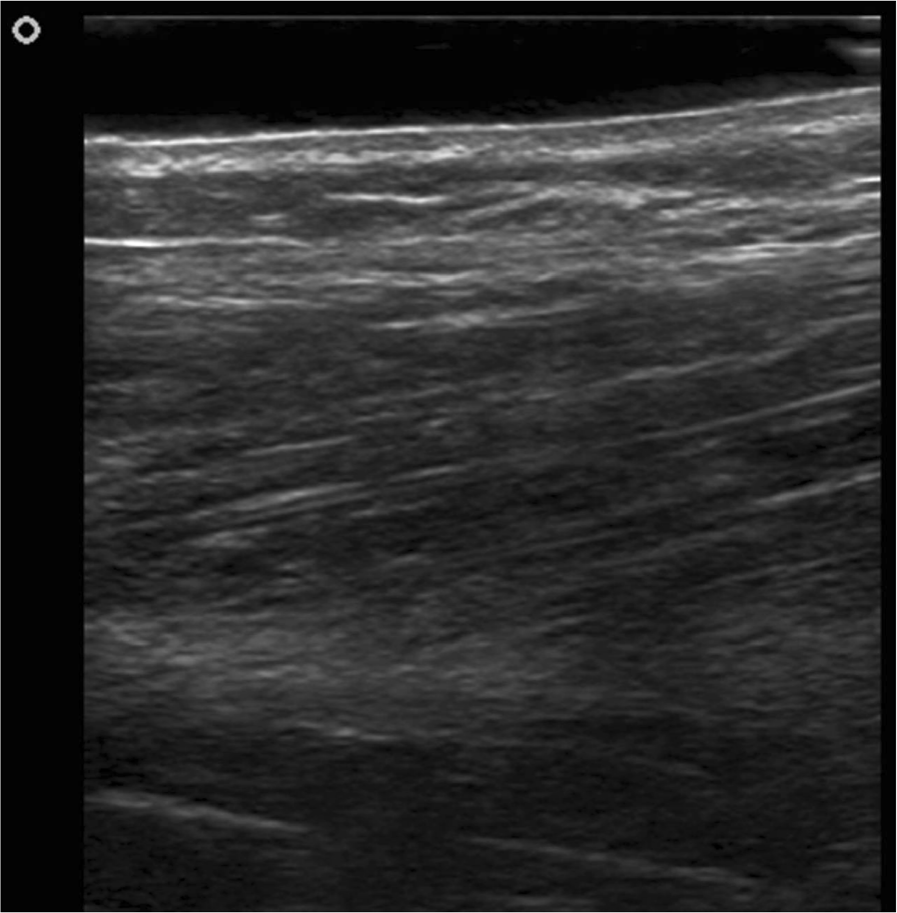
Ultrasound of anterior leg at rest is normal without any mass.

**Figure 2:**
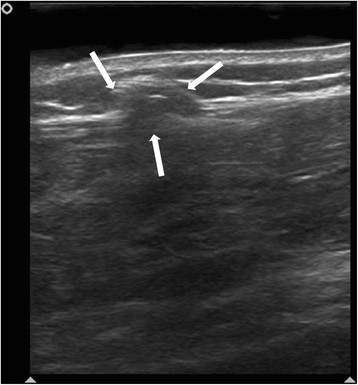
Ultrasound of the leg after the exercise shows 1 cm mass (white arrows) with fascial defect.

A 19-year-old female dancer was referred to our hospital after being evaluated as having a 1-cm nonpainful mass in her right anterior leg that appeared during the day and disappeared the next morning. The ultrasound result of her leg was negative for any pathology (Figure 1). The patient was asked to exercise her legs by going up and down the stairs of our six-story hospital for 10 min. At the end of this exercise, ultrasound showed a subcutaneous hypoechoic 1 cm mass (Figure 2 and Additional file 1) bulging out from the fascia of the tibialis anterior muscle with a defect in the fascia itself.

### Discussion

A muscular hernia through an overlying fascial defect, such as loosening or tearing, is most commonly reported in athletes or individuals with occupations that cause excessive strain on the legs like dancers such like our case. They occur usually in the lower limb, and usually, they affect the tibialis anterior muscle because its fascia is the most vulnerable to trauma, although other muscles in the lower leg, such as muscle of the extensor digitorum longus, peroneus longus, peroneus brevis, and gastrocnemius, have also been reported [1]. This rare entity could be acquired due to trauma or congenital due to fascial defect. Muscle hernia when present protrudes through a defect with the final result of subcutaneous soft tissue mass [2]. It was thought that isomeric muscle contraction in an upright position induces elevated pressure in fascias, and consequently, an elevated pressure causes muscle herniation at the weak points of fascias [3].

Clinical findings include the presence of soft, compressible nodules that vary in size depending on their position. This mass is characterized by herniation during leg dorsiflexion also known as ‘fencer's lunge position’ [4].

Although this condition is typically diagnosed by clinical observation, imaging specially ultrasound should confirm the diagnosis to avoid unnecessary biopsy. The clinical differential diagnoses are soft tissue tumors, such as lipoma, leiomyoma, Schwannoma, and dermatofibroma. However, these tumors do not change when a subject's position changes. The dynamic ultrasound study as shown in our case is essential in diagnosing anterior tibialis hernia. Usually, they are asymptomatic, but some person reported vague and fatigued pain aggravated by exercise and by work [1]–[4].

The treatment is controversial; surgery is an option in symptomatic patients. Dynamic ultrasound should be done in every patient suffering from leg pain or suffering from a strange vanishing behavior of leg masses. Static ultrasound could be negative as in our first ultrasound scan which was done during rest [5]. Ultrasound practitioners should ask the patients to exercise their muscles when muscle hernia is in the differential diagnosis.

## Conclusions

Anterior tibialis muscle hernia is a rare disease. Dynamic ultrasound is essential for determining the diagnosis. Ultrasound should be done after exercise of the affected limb.

## Consent

Written informed consent was obtained from the patient for publication of this case report and accompanying images. A copy of the written consent is available for review by the editor in chief of this journal.

## Competing interests

The authors declare that they have no competing interests.

## Authors' contributions

SA made the diagnosis and wrote the first draft. GH revised and approved the final draft. Both authors read and approved the final manuscript.

## Additional file

## Supplementary Material

Additional file 1: Dynamic ultrasound video clip of the leg after exercise shows the mass appearing and disappearing during dorsoflexion and extension of the foot. Click here for file
